# A review of human milk oligosaccharide concentrations of breast milk for infants and young children through 24 months of age

**DOI:** 10.3389/fped.2025.1649609

**Published:** 2025-09-04

**Authors:** Adam D. Kenney, Anice Sabag-Daigle, Mary-Margaret Stoecklein, Rachael H. Buck, Elizabeth J. Reverri

**Affiliations:** ^1^ Gut & Immunity Platform, Abbott Nutrition, Columbus, OH, United States; ^2^ Medical Safety & Surveillance, Abbott Nutrition, Columbus, OH, United States; ^3^ Nutrition Science & Innovation, Abbott Nutrition, Columbus, OH, United States

**Keywords:** human milk oligosaccharides, HMO, concentrations, review, meta-analysis

## Abstract

The World Health Organization and American Academy of Pediatrics both support continued breastfeeding beyond 12 months of age up to 24 months of age or beyond. Human milk oligosaccharides (HMOs) are the third most abundant solid component in breast milk. HMO concentrations in early breast milk have been well-characterized, but less is known about HMO profiles later in lactation. The goals of this literature review and meta-analysis of studies that analyzed HMO concentrations at 12 months of lactation or beyond were to identify the most abundant HMOs in breast milk at various timepoints throughout lactation and assess dynamic changes in HMO concentrations over time. Literature searches were conducted to identify studies on HMO quantification following PRISMA guidelines. Only studies that measured HMOs at/beyond one year of age were analyzed. In total, thirteen studies met eligibility criteria. The identity and number of HMOs measured in each study were recorded. HMOs that appeared in at least 10 articles, termed herein as core HMOs, were selected for further analysis. Concentrations of these HMOs, as well as total HMO levels, were grouped by timepoint (colostrum, 6-, 12-, and >12-months). Core HMOs were identified as 2′-fucosyllactose (2′-FL), 3-fucosyllactose (3-FL), lacto-N-tetraose (LNT), lacto-N-neotetraose (LNnT), 3′-sialyllactose (3′-SL), and 6′-sialyllactose (6′-SL). These HMOs accounted for >70% of the total HMO pool across timepoints. Total HMO concentrations decreased from colostrum to 6-months but plateaued at 12-months through 24 months. Individual core HMOs generally followed the same trend, with the exception of 3-FL, which increased in concentration over time through 12 months. Overall, HMO concentrations remained at significant levels through one year and the relative abundance of the core HMOs throughout lactation suggests biological relevance. Several studies have demonstrated associations between HMO concentrations in infants with outcomes in young children. Extending these analyses to include prolonged consumption of HMOs (> one year) would be of general interest to the field. To the best of our knowledge, this review is the first to specifically synthesize studies that analyzed HMO concentrations at 12 months lactation. Further research may enhance the understanding of the effects of HMOs beyond infancy and into young childhood.

## 1 Introduction

Breastfeeding is the gold standard of feeding for infants. The American Academy of Pediatrics (AAP) recently published their updated United States (US) policy statement that supports continued breastfeeding to two + years of age, as long as mutually desired by mother and child, with introduction of complementary foods at approximately six months of age (1). This aligns with global breastfeeding recommendations by the World Health Organization (WHO) that emphasize breast milk is an important source of energy and nutrients in the second year of life, providing about one-third of energy needs, and may reduce mortality in malnourished children (2). Despite these breastfeeding recommendations, only 35.9% of young children in the US are breastfed up to 12 months of age with no further data points reported (3). Worldwide, approximately 70% of young children are breastfed up to 12 months of age, which decreases to 45% by 24 months of age (4).

In breast milk, human milk oligosaccharides (HMOs) are the third most abundant solid component. HMO concentrations have been well-characterized from breast milk of mothers of infants (5–8). HMOs have been shown to have multi-functional benefits both preclinically (9) and clinically (10), including in infants fed formula with added HMO (11). Moreover, several studies from within the past decade have shown correlations between HMO consumption from breast milk in infancy and positive outcomes later in childhood (12–20). However, associations between extended consumption of HMOs beyond infancy and later outcomes have largely been unexplored to date. In general, young child nutrition is an understudied area (21). Adding to this dearth of information, few studies have empirically investigated HMO concentrations beyond the first 3–6 months of lactation, and in those that have, measuring HMO concentrations at later timepoints has often not been the primary outcome. In fact, in a recent comprehensive review that compiled 57 studies in which HMO concentrations were measured throughout lactation, only two of the studies included measurements at 12 months of age (7). Further, the authors of that review aggregated all data from measurements occurring beyond 90 days of lactation into one category, underscoring how later measurement timepoints may be obscured within heterogenous datasets.

In consideration of these points, we aimed to specifically compile studies in which HMO concentrations were analyzed at 12 months of age and beyond in order to assess dynamic changes in HMO concentrations at discrete timepoints later in lactation. To our knowledge, this review and meta-analysis is the first of its kind to summarize HMO concentrations in breast milk with a precise focus on timepoints beyond early infancy.

## 2 Materials and methods

**Figure 1:**
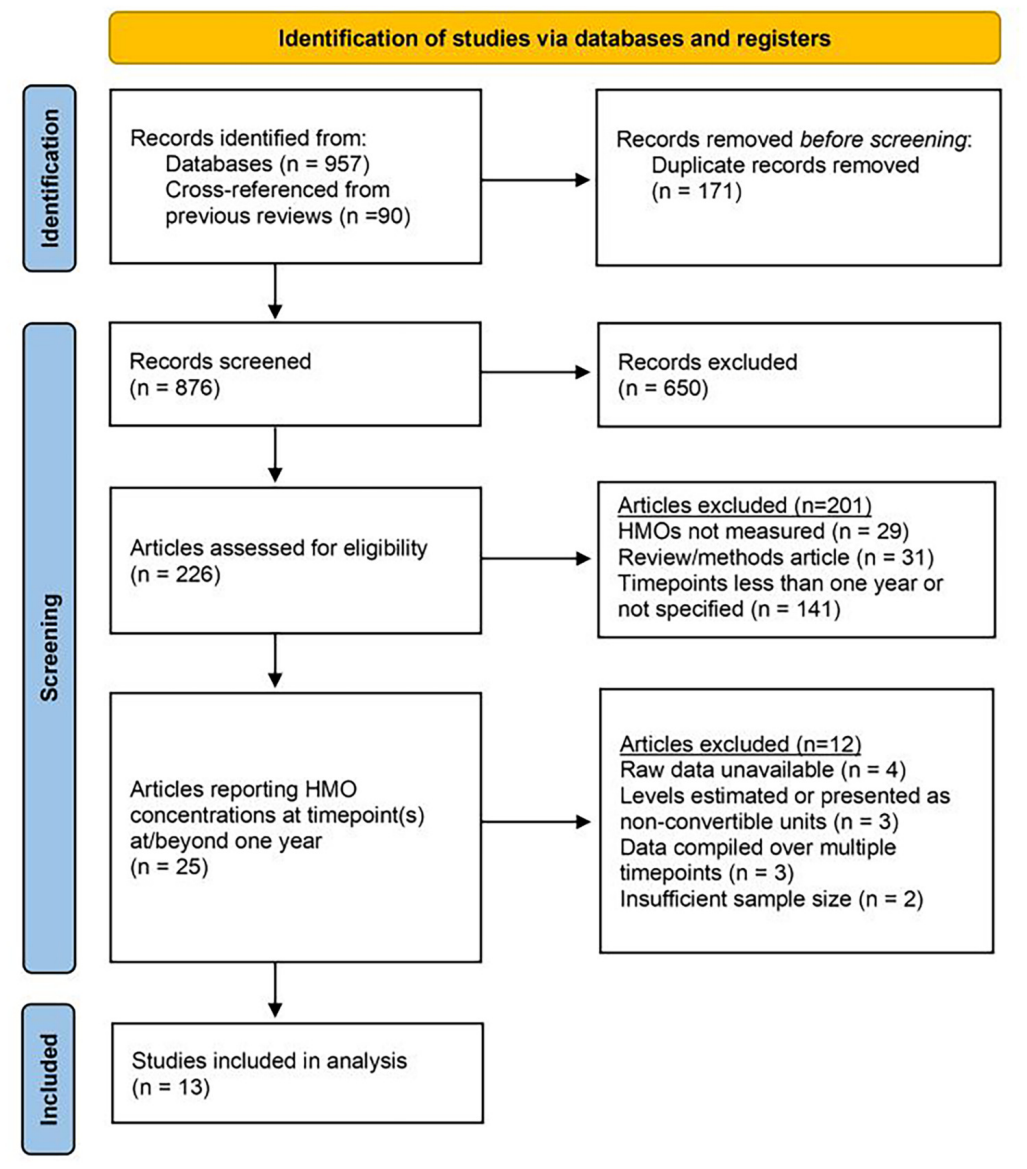
PRISMA workflow. A total of 876 unique articles were screened. 650 that were obviously out-of-scope were excluded prior to retrieval. 201 articles were excluded via primary exclusion criteria. An additional 12 articles that measured HMOs at 12 months were excluded based on secondary exclusion criteria. 13 studies were included for review and analysis. PRISMA workflow template reproduced with permission under Creative Commons Attribution License (CC BY 4.0) (35, 36).

Literature searches, concluding on January 31st, 2025, were conducted on PubMed and Google Scholar, with a combinations of search terms employed (human milk oligosaccharide(s) one year; human milk oligosaccharide(s) 12 months; human milk oligosaccharide(s) concentration; human milk oligosaccharide(s) quantification). Additionally, three previous literature reviews (5–7) assessing HMO concentrations were cross-referenced for additional articles. The main inclusion criterion was as follows: only studies that measured HMO concentrations in at least one instance at or beyond one year (or an approximation thereof) were considered for inclusion. Where applicable, earlier measurement timepoints from these specific studies were used as reference points in the downstream data analysis. Additionally, only peer-reviewed articles published in or translated to English which had available datasets that could be reasonably obtained were included. Exclusion was performed independently by two authors (ADK and ASD). The full PRISMA workflow used for article exclusion can be found in Figure 1. In total, 13 research articles met all inclusion criteria (22–34). Key characteristics of these studies are represented in Table 1.

**Table 1:** Overview of articles included in the literature review. Articles are listed chronologically.

Ref.	Sample size	Country	Secretor status definition	Measurement timepoints (postpartum)	HMOs measured	No. of HMOs measured	Analysis method
(22)	435 mothers	Chile, China, France, Italy, Mexico, USA, Philippines, Singapore, Sweden	Not Determined	0–2, 3–10, 11–30, 31–452 days	2′-FL, 3-FL, DFL, LNT, LNnT, LNFP-I, LNFP-II, LNFP-III, LNFP-V	9	High-performance anion-exchange chromatography (HPAEC)
(23)	131 longitudinal breast milk samples from 19 mothers and 33 unpasteurized, pooled human milk samples from Mother's Milk Bank	USA	Not Determined	9–17 months	Total of: 2′-FL, 3-FL, 3′-SL, 6′-SL, LNT, LNFP-I, LNFP-II, LNFP-III, LDFT, LNDFH-I, LNDFH-II, DSLNT, SLNFP-II, MNT, SLNFP-IV, MNnT	Total of 16 HMOs	Carbon high-performance liquid chromatography with tandem mass spectrometry (HPLC-MS)
(24)	26 mothers	Malaysia	Not Determined	Colostrum (<5 days), 60, 180, 365 days	2′-FL, 3-FL, 3′-SL, 6′-SL, DSL, LNT, LNnT, 6′-SLN, 3′-S3FL, LSTa/b, LSTc, Total LNFP	12	Hydrophilic interaction liquid chromatography with mass spectrometry (HILIC-MS)
(25)	*n* = 156 (3 months) *n* = 122 (6 months) *n* = 28 (12 months)	Germany	FUT2 SNP rs601338	3, 6, 12 months	2′-FL, 3-FL, 3′-SL, 6′-SL, LNT, LNnT, LNFP-I, LNFP-II, LNFP-III, LNFP-V, LDFT, LNDFH-I, 3′-GL, 6′-GL, LSTb, LSTc, LNnDFH, LNnFP-V, A-tetra, DSLNT, DFLNHa, LNH, MFLNH-III Hex4 HexNAc2	24	Liquid chromatography with mass spectrometry (LC-MS)
(26)	*n* = 207 (1 month) *n* = 109 (6 months) *n* = 83 (12 months) *n* = 59 (18 months) *n* = 28 (24 months)	USA	Presence or near absence of 2′-FL (<100 nmol/ml)	1, 6, 12, 18, 24 months	2′-FL, 3-FL, DFL, 3′-SL, 6′-SL, LNT, LNnT, LNFP-I, LNFP-II, LNFP-III, LNH, DFLNHa, DFLNT, DSLNH, DSLNT, LSTb, LSTc, FDSLNH, FLNH	19	HPLC
(27)	*n* = 682 (6 weeks) *n* = 448 (6 months) *n* = 73 (12 months)	Germany	Defined but HMO levels were not stratified by group	6 weeks, 6 months, 12 months	2′-FL, 3-FL, DFL, 3′-SL, 6′-SL, LNT, LNnT, LNFP-I, LNFP-II, LNFP-III, LNFP-V, LNDFH-I, LNDFH-II, LNnDFH II, 6′-GL	15	Targeted liquid chromatography electrospray ionization with tandem mass spectrometry (LC-ESI-MS)
(28)	488 samples from 335 mothers	China	Not Determined	0–5, 10–15, 40–45, 200–240, 300–400 days	2′-FL, 3-FL, 3′-SL, 6′-SL, LNT, LNnT	6	High-performance anion-exchange chromatography- pulsed ampero-metric detector (HPAEC-PAD)
(29)	167 samples from 71 mothers	United Kingdom	FUT2 SNP rs516246 G/G and A/G alleles = secretor A/A allele = non-secretor	Birth, 2 weeks, 6 weeks, 3 months, 6 months, 12 months	2′-FL, 3-FL, 3′-SL, 6′-SL, LNT, LNnT, LNFP-I	7	HPAEC
(30)	383 samples from 277 mothers *n* = 100 (0–5 days) *n* = 20 (10–15 days) *n* = 91 (40–45 days) *n* = 93 (200–240 days) *n* = 79 (300–400 days)	China	Presence of 2′-FL >200 mg/L	0–5, 10–15, 40–45, 200–240, 300–400 days	2′-FL, 3-FL, 3′-SL, 6′-SL, LNT, LNnT	6	HPAEC-PAD
(31)	481 milk samples (random sub- sample from larger study)	China	Levels of 2′-FL, LDFT, LNFP-I, and LNnDFH-I	Colostrum (0–6 days), Transitional milk (7–14 days), Mature milk (15–340 days)	2′-FL, 3-FL, 3′-SL, 6′-SL, LNT, LNnT, LNFP-I, LNFP-II, LNFP-III, DSLNT, LDFT, LSTb, LSTc, [LNDFH-I + LNnDFH-I], LNDFH-II, LNnDFH-II, MFLNH-I, MFLNH-III, MFLNnH, DFLNHa, DFpLNnH, [3′-SLNFPII + 6′-SLNFPVI]	22	Ultra-high performance liquid chromatography with mass spectrometry and multi-reaction monitoring (UPLC-MRM)
(32)	1,758 mothers	China	Low and high 2′-FL noted	0–5, 10–15, 40–45, 200–240, 300–400 days	2′-FL, 3-FL, 3′-SL, 6′-SL, LNT, LNnT	6	HPAEC-PAD
(33)	Longitudinal breast milk samples from 210 mothers *n* = 210 (1 month) *n* = 131 (6 months) *n* = 84 (12 months)	USA	Not Determined	1, 6, 12 months	2′-FL, 3-FL, 3′-SL, 6′-SL, DFL, LNT, LNnT, LNFP-I, LNFP-II, LNFP-III, DFLNT, DSLNT, LNH, FLNH, DFLNH, FDSLNH, DSLNH, LSTb, LSTc	19	HPLC
(34)	129 mothers *n* = 36 (0–3 months) *n* = 33 (3–6 months) *n* = 43 (6–9 months) *n* = 15 (9–12 months)	China	Not Determined	0–3, 3–6, 6–9, 9–12 months	2′-FL, 3-FL, 3′-SL, 6′-SL	4	Nuclear magnetic resonance (*N*MR)

**Figure 2:**
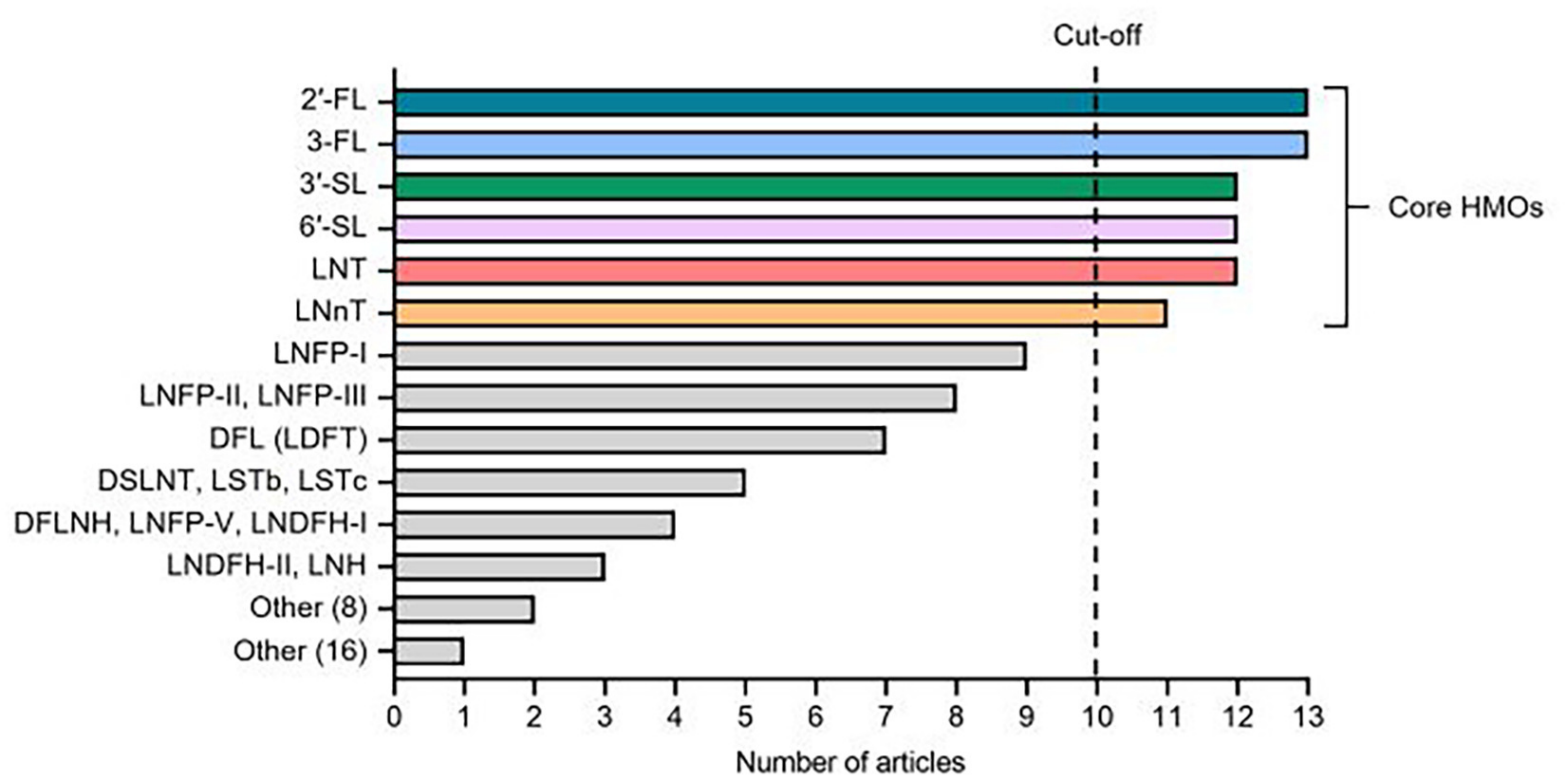
Identity and frequency of individual HMOs analyzed in the 13 studies included in the literature review. 42 distinct HMOs were measured at least once across the studies. Core HMOs are defined as those appearing in at least 10 articles. Other (8) includes DSLNH, DFLNT, LNnDFH-I, LNnDFH-II, FLNH, FDSLNH, MFLNH-III, and 6′-GL. Other (16) includes DSL, DFpLNnH, LNnFP-V, SLNFP-II, SLNFP-IV, MFLNH-I, MFLNnH, MNT, MNnT, 3′-GL, 3-S3FL, 6SLN, [3SLNFP-II + 6SLNFP-VI], LSTa, Hex4 HexNAc2, and A-Tetra.

Identities of the individual HMOs and total number of HMOs measured in each study were recorded. For each unique HMO, the measurement frequency was calculated from a total possible inclusion score of 13 (Figure 2). HMOs that appeared in a minimum of ten articles, termed herein as core HMOs, were selected for further analy-sis and were as follows: 2′-fucosyllactose (2′-FL), 3-fucosyllactose (3-FL), 3′-sialyllactose (3′-SL), 6′-sialyllactose (6′-SL), lacto-N-tetraose (LNT), and lac-to-N-neotetraose (LNnT). Concentrations of these six core HMOs were extracted from the available datasets and supplemental materials and compiled by selected timepoints of interest (colostrum and 6 months of age as reference timepoints, 12 months of age, and all timepoints >12 months up to 24 months of age).

Additional curation of the data prior to analysis included standardizing all data-points to be presented as g/L and calculating total HMO levels at each timepoint (when not explicitly provided in the individual studies) by summing the mean values of all HMOs measured in that study. In the case of Mokhtari et al., conversion from nmol/ml to g/L could not reliably be performed for all HMOs measured, since molecular mass data could not be obtained for DFLNT and FDSLNH. As such, individual HMO concentrations were assessed for the core HMOs, but total concentrations were not calculated from this study (33). Further, necessary binning of timepoints that were not explicitly defined based on the above definitions was performed. For example, samples that were not defined explicitly as colostrum but that were measured on or before five days lactation were also considered to be colostrum samples. In some instances, the closest approximation to the defined timepoints was used, such as HMO concentrations measured at 180 days being included in the 6 months of age group, or values from a timepoint of “300–400 days” being included in the 12 months of age group. In general, reasonable timepoint approximations were used wherever possible to avoid further narrowing of the dataset. Finally, data striated based on secretor status were treated as separate datapoints, where applicable. Concentrations of 2′-FL from non-secretor samples were excluded from the analysis, as these values were near zero, consistent with findings that 2′-FL is largely undetectable in milk from non-secretors (37, 38).

**Figure 3:**
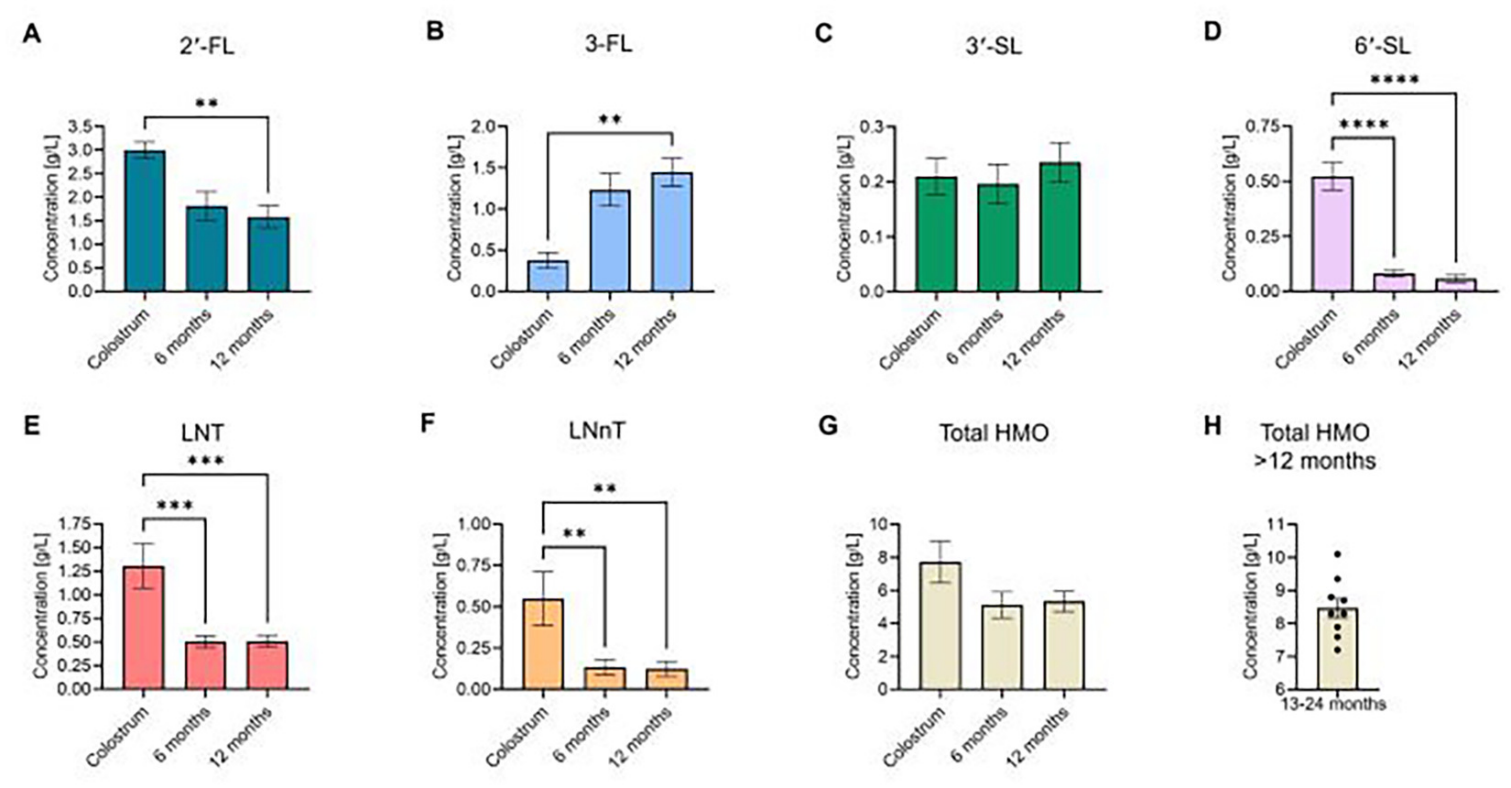
Concentrations of six core and total HMOs in breast milk. Mean values of the six most commonly measured HMOs across the studies [**(A)** 2′-FL, **(B)** 3-FL, **(C)** 3′-SL, **(D)** 6′-SL, **(E)** LNT, **(F)** LNnT], as well as **(G)** total HMO levels, were compiled based on timepoint: colostrum, 6 months, and 12 months. **(H)** > 12 months of age timepoints (total HMO levels only; aggregate data of HMO levels collected between 13 and 24 months lactation). Data were extracted from the provided data tables or supplemental materials in each study. Total HMO levels were either provided in the individual studies or were calculated by summing the mean values of all HMOs measured in that specific study. Bars represent the mean-of-means for individual HMOs/timepoints across all studies. HMO concentrations across the included timepoints were assessed for statistically significant changes via one-way ANOVA with Tukey's *post-hoc* multiple comparisons test. *P < 0.05; ** *P* < 0.01; ***P < 0.001; ****P < 0.0001.

**Figure 4:**
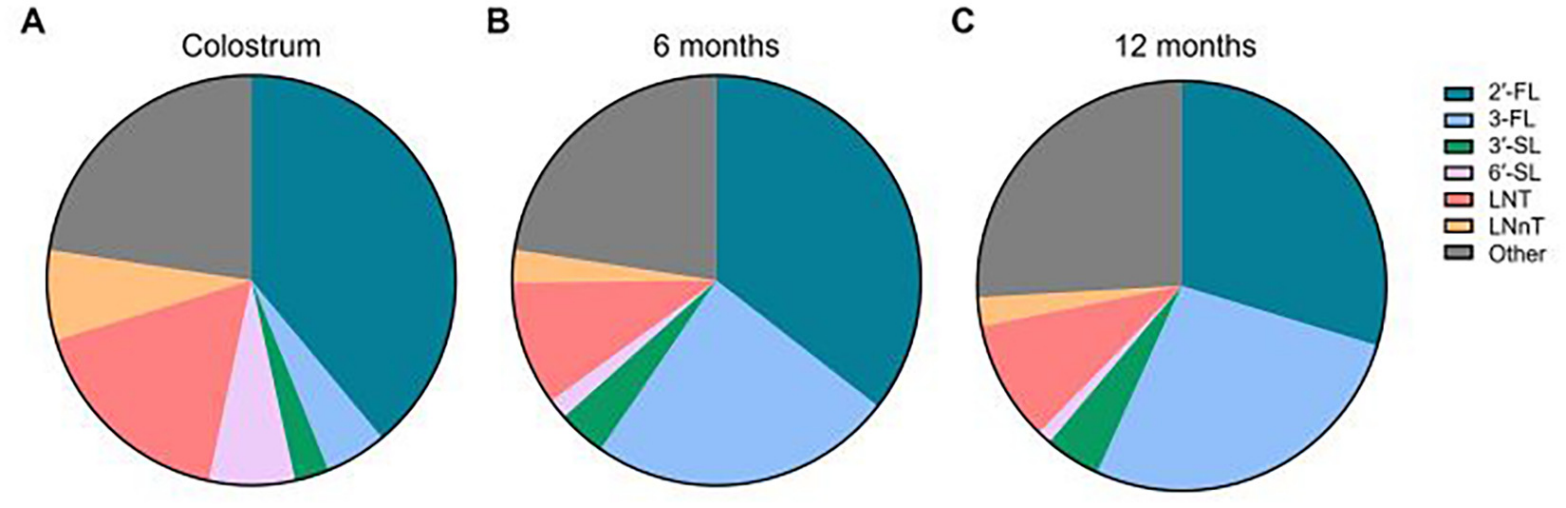
Relative abundance of the six core HMOs in breast milk between 0 and 12 months of age. Ratios of the six individual HMOs as in Figure 3 were calculated in relation to total HMO concentration. Ratios were calculated by taking the quotient of average individual HMO levels divided by average total HMO level at the respective timepoint and multiplying by 100%. “Other” was calculated as 100% - [Sum % of 2′-FL, 3-FL, 3′-SL, 6′-SL, LNT, LNnT]. **(A)** Colostrum, **(B)** 6 months, **(C)** 12 months.

The compiled values for the six core HMOs, as well as total HMO concentrations, across these 13 studies were then graphed, with bars representing the overall mean-of-means and error bars representing standard deviation (Figure 3, Table 2). From this analysis, relative proportions of these HMOs in comparison to total HMO levels at each timepoint were calculated by taking the quotient of individual HMO averages divided by the total HMO average and multiplying by 100%, with “other” being calculated as 100% - [Sum % of 2′-FL, 3-FL, 3′-SL, 6′-SL, LNT, LNnT] (Figure 4, Table 2).

**Table 2:** Concentration and relative abundance of the six core HMOs at 0–12 months of age across the 13 studies analyzed.

HMO	Timepoint					
	Colostrum		6mo		12mo	
	Concentration (g/L)	Relative abundance (%)	Concentration (g/L)	Relative abundance (%)	Concentration (g/L)	Relative abundance (%)
2′-FL	3.00	*38.9*	1.82	*35.5*	1.59	*29.7*
3-FL	0.38	*4.9*	1.23	*24.1*	1.45	*27.1*
LNT	1.31	*16.9*	0.50	*9.8*	0.50	*9.5*
LNnT	0.55	*7.1*	0.13	*2.6*	0.12	*2.3*
3′-SL	0.21	*2.7*	0.20	*3.8*	0.24	*4.4*
6′-SL	0.52	*6.8*	0.08	*1.6*	0.06	*1.1*
Total	*7.72*	—	5.12	—	5.34	—

Italicized values represent relative abundance (%) of the specified individual HMO in comparison to total HMO concentration at each timepoint. These values are represented graphically in Figure 4.

## 3 Results

In total, 42 unique HMOs were measured at least once across the 13 articles, with over a third of these HMOs (38.1% or 16 of 42 HMOs) only appearing in one instance across all studies. Only ten HMOs were represented in at least half of the included studies. The six core HMOs selected for further analysis, defined above as those that were analyzed in at least ten studies, included 2′FL, 3-FL, 3′-SL, 6′-SL, LNT, and LNnT. One study (23) reported total HMO levels based on 16 HMOs, including 2′-FL, 3-FL, 3′-SL, 6′-SL and LNT. Only 2′-FL and 3-FL were measured in every study. The identity of HMOs measured and the number of articles in which they appear are summarized in Figure 2.

The most common timepoints where HMOs were measured in these studies were in colostrum and at 6- and 12-months of age. Most studies concluded measurements at one year. Only Perrin et al. (23) (monthly measurements of total HMO levels up to 17 months of age) and Plows et al. (26) (individual concentrations of 19 HMOs at 18- and 24-months of age) measured HMOs at appreciably later timepoints. As such, our analysis of individual HMO levels was capped at 12 months of age, with colostrum and 6-month timepoints serving as reference points from which changes in HMO concentrations could be assessed. We included an additional timepoint (>12 months of age) for total HMO concentrations which represents aggregated data from *n* = 9 datapoints, comprising all measurements from 13 to 24 months of age in two studies (23, 26).

Our analysis revealed that 2′-FL was the most abundant HMO in breastmilk across all timepoints (Figure 3A, Table 2). At an average concentration of 3.00 g/L in colostrum, this was more than double the level of the next most abundant HMO, which was LNT at 1.31 g/L (Figures 3A,E, Table 2). Moreover, despite a significant decline in concentration over the course of lactation, 2′-FL remained slightly more abundant at 12 months compared to 3-FL (1.59 g/L vs. 1.45 g/L) (Figures 3A,B, Table 2). These data align with previous observations that 2′-FL is the most prominent breastmilk oligosaccharide (7). Inversely, 3-FL, the second most abundant fucosylated oligosaccharide in breastmilk, was the only HMO found to significantly increase over time (0.38 g/L in colostrum vs. 1.45 g/L at 12 months, a nearly four-fold increase) (Figure 3B, Table 2). The remaining HMOs that were analyzed either decreased from colostrum to 12 months of age (6′-SL, LNT, and LNnT) or plateaued (3′-SL) (Figures 3C–F, Table 2). Total HMO concentrations also declined throughout lactation (7.72 g/L in colostrum to 5.34 g/L at 12 months) (Figure 3G, Table 2). HMO concentrations from all timepoints >12 months of age ranged from 7.2 g/L to 10.1 g/L, with an average concentration of 8.47 g/L (Figure 3H). These data are plotted separately given that all datapoints originated from only two studies, making statistical comparisons to previous timepoints not possible. Proportionally, the six core HMOs accounted for more than 70% of the total HMO pool at each timepoint (Figures 4A–C, Table 2).

## 4 Discussion

Whereas the breadth of literature investigating breast milk HMO levels during the infancy year is extensive and diverse, the number of studies analyzing at or beyond 12 months of age is comparatively limited. As previously mentioned, Soyyilmaz et al. recently published an extensive review covering HMO levels throughout lactation, amounting to 57 publications (7). In contrast, the dataset included herein is derived from a pool of literature <25% of that scale despite less stringent inclusion criteria, underscoring that the focus in the field has been predominantly early lactation. However, the cumulative sample size (*n* = 4,786) across all studies included was still quite robust. Moreover, the authors of that study aggregated all data from measurements occurring be-yond 90 days of lactation into one category (7). Nonetheless, the findings reported here are largely in agreement with what has been previously published on HMO levels in early milk. Specifically, the relative abundance of selected HMOs and trends in total HMO levels (overall decline) over time were consistent out to 12 months of age (Figure 3). Of note, there was some minor variation between the relative abundance of the core HMOs reported here and what has been described previously. This can likely be attributed to the aforementioned smaller sample size of studies included in this review, which precluded further analysis of additional predominant HMOs, such as LNFP-I and LNDFH-I. Interestingly, the aggregated values for total HMO levels from all >12 months of age timepoints represented the highest average concentration of any timepoint. However, because these datapoints were compiled from a wide range of collection timepoints across a much smaller pool of studies, any meaningful comparison should be drawn with caution until more substantive analysis can be completed from a larger pool of data which may become available from future studies.

There are several other key considerations that may further shape interpretation of the data presented herein. In an analysis comprising so few studies and in which there exists disparity in analysis methods, experimental design, and data reporting, intra-study variation will inherently lend greater influence on the overall results. Specific factors that could not be controlled for here, due to the necessarily lenient exclusion criteria, include HMO analytical method, definition and striation of secretor vs. non-secretor milk, geographic location of the studies, relative sample size consistency across timepoints, health of the mother/infant dyads, and birth/delivery mode of the infant. For instance, the studies that utilized HPLC tended to quantify a more diverse set of HMOs (12–24 HMOs) compared to those that used HPAEC (6–9 HMOs), both of which were greater than the one study that employed NMR analysis (4 HMOs) (Table 1). Even within conserved methods across studies, there was considerable variation in the data reported. Moreover, since the studies represented herein analyzed a variety of HMOs, it stands to reason that the most commonly measured core HMOs may have been overrepresented in studies where these HMOs comprised most, or all, of the total HMO pool measured in that particular study (Figure 4). This may also be an example of confirmation bias, wherein these HMOs have historically been considered among the most abundant HMOs in breastmilk (10, 39, 40) and therefore emphasis was placed on including them in the analysis (Figure 2). Additionally, secretor status of the mother is well-established to disproportionately affect levels of certain HMOs, namely 2′-FL (37, 38, 41), yet secretor status was only considered for data presentation in five studies, with variable definitions for determining secretor vs. non-secretor in each (Table 1). Likewise, impact of geographical origin of the samples on HMO concentrations was not considered here despite disparate sampling across the United States, Asia, and Europe, although previous reports have suggested that there are significant differences in HMO levels across the globe (42). Where sample size was reported by individual timepoint in contrast to overall sample size, late timepoint *n* numbers were consistently the lowest values and in some cases were nearly 10-fold lower compared to early timepoints (Table 1), which may have introduced further variability within certain portions of the datasets. Indeed, even from six to 12 months of age, cumulative sample size from this subset of studies decreased from *n* = 936 at six months of age to *n* = 362 at 12 months of age, representing a 61% reduction in the number of mothers represented. Other factors, such as health of the mother/infant dyads and birth/delivery mode, were often not reported within the studies at all, although the contribution of these factors to breastmilk HMO concentrations is unclear. Taken together, these considerations underscore the inherent difficulty in drawing overarching conclusions about HMO levels at later timepoints in lactation and highlight a key knowledge gap in the field which warrants further study. Nonetheless, the results herein highlight the significant contributions of the so-called core HMOs to the overall HMO pool throughout lactation and suggest that total HMO levels remain at biologically significant levels through the first two years of breastfeeding.

Interestingly, only one study included in this review was published prior to the last decade. The remaining studies were published between 2017-present, potentially indicating a shift in interest/focus to HMO levels beyond one year, in alignment with updated US breastfeeding guidelines that support breastfeeding beyond one year (1, 2). Despite this recent attention, more research focused specifically on HMO concentrations beyond one year of lactation is warranted. Intriguingly, an increasing number of breast milk association studies have been published relating HMO concentrations in breast milk during infancy to positive outcomes into young childhood (12–20). Extending these analyses to include prolonged consumption of HMOs up to two years would be valuable to the field. Additionally, if a young child is not receiving breast milk, HMOs may be provided by select young child formulas/toddler drinks and/or dietary supplements intended for young children, when warranted.

## References

[1] Meek JY Noble L, Section on Breastfeeding. Policy statement: breastfeeding and the use of human milk. Pediatrics. (2022) 150:e2022057988. 10.1542/peds.2022-057988 35921640

[2] WHO. Infant and young child feeding. (2023).

[3] CDC. Breastfeeding Report Card. (2024).

[4] UNICEF/WHO. GLOBAL BREASTFEEDING SCORECARD 2023. (2023).

[5] Thurl S Munzert M Boehm G Matthews C Stahl B. Systematic review of the concentrations of oligosaccharides in human milk. Nutr Rev. (2017) 75:920–33. 10.1093/nutrit/nux044 29053807 PMC5914348

[6] Thum C Wall CR Weiss GA Wang W Szeto IMY Day L. Changes in HMO concentrations throughout lactation: influencing factors, health effects and opportunities. Nutrients. (2021) 13:2272. 10.3390/nu13072272 34209241 PMC8308359

[7] Soyyılmaz B Mikš MH Röhrig CH Matwiejuk M Meszaros-Matwiejuk A Vigsnæs LK. The mean of milk: a review of human milk oligosaccharide concentrations throughout lactation. Nutrients. (2021) 13:2737. 10.3390/nu13082737 34444897 PMC8398195

[8] Urashima T Ajisaka K Ujihara T Nakazaki E. Recent advances in the science of human milk oligosaccharides. BBA Adv. (2025) 7:100136. 10.1016/j.bbadva.2024.100136 39991261 PMC11847054

[9] Hill DR Chow JM Buck RH. Multifunctional benefits of prevalent HMOs: implications for infant health. Nutrients. (2021) 13:3364. 10.3390/nu13103364 34684364 PMC8539508

[10] Bode L. Human milk oligosaccharides: every baby needs a sugar mama. Glycobiology. (2012) 22:1147–62. 10.1093/glycob/cws074 22513036 PMC3406618

[11] Schonknecht YB Moreno Tovar MV Jensen SR Parschat K. Clinical studies on the supplementation of manufactured human milk oligosaccharides: a systematic review. Nutrients. (2023) 15:3622. 10.3390/nu15163622 37630811 PMC10458772

[12] Berger PK Bansal R Sawardekar S Yonemitsu C Furst A Hampson HE Associations of human milk oligosaccharides with infant brain tissue organization and regional blood flow at 1 month of age. Nutrients. (2022) 14:3820. 10.3390/nu14183820 36145194 PMC9501015

[13] Berger PK Ong ML Bode L Belfort MB. Human Milk Oligosaccharides and Infant Neurodevelopment: A Narrative Review. Nutrients. (2023) 15:719. 10.3390/nu15030719 36771425 PMC9918893

[14] Berger PK Plows JF Jones RB Alderete TL Yonemitsu C Poulsen M Human milk oligosaccharide 2'-fucosyllactose links feedings at 1 month to cognitive development at 24 months in infants of normal and overweight mothers. PLoS One. (2020) 15:e0228323. 10.1371/journal.pone.0228323 32049968 PMC7015316

[15] Oliveros E Martín MJ Torres-Espínola FJ Segura-Moreno MT Ramírez M Santos A Human milk levels of 2-fucosyllactose and 6-sialyllactose are positively associated with infant neurodevelopment and are not impacted by maternal BMI or diabetic status. J Nutr Food Sci. (2021) 4:100024.

[16] Sato K Nakamura Y Fujiyama K Ohneda K Nobukuni T Ogishima S Absolute quantification of eight human milk oligosaccharides in breast milk to evaluate their concentration profiles and associations with infants’ neurodevelopmental outcomes. J Food Sci. (2024) 89:10152–70. 10.1111/1750-3841.17597 39656795 PMC11673463

[17] Cho S Zhu Z Li T Baluyot K Howell BR Hazlett HC Human milk 3'-sialyllactose is positively associated with language development during infancy. Am J Clin Nutr. (2021) 114:588–97. 10.1093/ajcn/nqab103 34020453 PMC8326052

[18] Mansell T Furst A O'Hely M Chang M Ponsonby A-L Vuillermin P Age-dependent associations of human milk oligosaccharides with body size and composition up to 4 years of age. Am J Clin Nutr. (2023) 117:930–45. 10.1016/j.ajcnut.2023.02.016 36813025 PMC10447468

[19] Wejryd E Freiholtz Jern E Marchini G Åden U Landberg E Abrahamsson T. Human milk oligosaccharides in breast milk at two weeks of age in relation to neurodevelopment in 2-year-old children born extremely preterm. An Explorative Trial. Nutrients. (2025) 17:832. 10.3390/nu17050832 40077703 PMC11902041

[20] Sawane K Takahashi I Ishikuro M Takumi H Orui M Noda A Association between human milk oligosaccharides and early adiposity rebound in children: a case-control study of the tohoku medical megabank project birth and three-generation cohort study. J Nutr. (2025) 155:1498–507. 10.1016/j.tjnut.2025.02.024 40058699

[21] Reverri EJ Arensberg MB Murray RD Kerr KW Wulf KL. Young child nutrition: knowledge and surveillance gaps across the Spectrum of feeding. Nutrients. (2022) 14:3093. 10.3390/nu14153093 35956275 PMC9370290

[22] Erney RM Malone WT Skelding MB Marcon AA Kleman-Leyer KM O'Ryan ML Variability of human milk neutral oligosaccharides in a diverse population. J Pediatr Gastroenterol Nutr. (2000) 30:181–92. 10.1097/00005176-200002000-00016 10697138

[23] Perrin MT Fogleman AD Newburg DS Allen JC. A longitudinal study of human milk composition in the second year postpartum: implications for human milk banking. Matern Child Nutr. (2017) 13:e12239. 10.1111/mcn.12239 26776058 PMC6866067

[24] Ma L McJarrow P Mohamed HJB Liu X Welman A Fong BY. Lactational changes in the human milk oligosaccharide concentration in Chinese and Malaysian mothers’ milk. Int Dairy J. (2018) 87:1–10. 10.1016/j.idairyj.2018.07.015

[25] Lefebvre G Shevlyakova M Charpagne A Marquis J Vogel M Kirsten T Time of lactation and maternal fucosyltransferase genetic polymorphisms determine the variability in human milk oligosaccharides. Front Nutr. (2020) 7:574459. 10.3389/fnut.2020.574459 33195368 PMC7658960

[26] Plows JF Berger PK Jones RB Alderete TL Yonemitsu C Najera JA Longitudinal changes in human milk oligosaccharides (HMOs) over the course of 24 months of lactation. J Nutr. (2021) 151:876–82. 10.1093/jn/nxaa427 33693851 PMC8030713

[27] Siziba LP Mank M Stahl B Gonsalves J Blijenberg B Rothenbacher D Human milk oligosaccharide profiles over 12 months of lactation: the ulm SPATZ health study. Nutrients. (2021) 13:1973. 10.3390/nu13061973 34201331 PMC8228739

[28] Liu S Cai X Wang J Mao Y Zou Y Tian F Six Oligosaccharides’ variation in breast milk: a study in south China from 0 to 400 days postpartum. Nutrients. (2021) 13:4017. 10.3390/nu13114017 34836272 PMC8623037

[29] Durham SD Robinson RC Olga L Ong KK Chichlowski M Dunger DB A one-year study of human milk oligosaccharide profiles in the milk of healthy UK mothers and their relationship to maternal FUT2 genotype. Glycobiology. (2021) 31:1254–67. 10.1093/glycob/cwab057 34142145

[30] Li X Mao Y Liu S Wang J Li X Zhao Y Vitamins, vegetables and metal elements are positively associated with breast milk oligosaccharide composition among mothers in Tianjin, China. Nutrients. (2022) 14:4131. 10.3390/nu14194131 36235783 PMC9570563

[31] Ren X Yan J Bi Y Shuttleworth PW Wang Y Jiang S Human milk oligosaccharides are associated with lactation stage and lewis phenotype in a Chinese population. Nutrients. (2023) 15:1408. 10.3390/nu15061408 36986137 PMC10059825

[32] Liu S Mao Y Wang J Tian F Hill DR Xiong X Lactational and geographical variation in the concentration of six oligosaccharides in Chinese breast milk: a multicenter study over 13 months postpartum. Front Nutr. (2023) 10:1267287. 10.3389/fnut.2023.1267287 37731395 PMC10508235

[33] Mokhtari P Schmidt KA Zamanian H Babaei M Machle CJ Trifonova D Maternal diet associated with oligosaccharide abundances in human milk from Latina mothers. Nutrients. (2024) 16:1795. 10.3390/nu16121795 38931150 PMC11206877

[34] Chen G Chen L Wang H Zhang J Sun X Chen X (1)H nuclear magnetic resonance-based metabolomic profiling and comparison of human milk across different lactation stages in secretors and nonsecretors: a study of Chinese lactating women. J Nutr. (2025) 155:78–86. 10.1016/j.tjnut.2024.10.050 39491676

[35] Page MJ McKenzie JE Bossuyt PM Boutron I Hoffmann TC Mulrow CD The PRISMA 2020 statement: an updated guideline for reporting systematic reviews. BMJ. (2021) 372:n71. 10.1136/bmj.n71 33782057 PMC8005924

[36] Page MJ Moher D Bossuyt PM Boutron I Hoffmann TC Mulrow CD PRISMA 2020 explanation and elaboration: updated guidance and exemplars for reporting systematic reviews. BMJ. (2021) 372:n160. 10.1136/bmj.n160 33781993 PMC8005925

[37] Grollman EF Ginsburg V. Correlation between secretor status and the occurrence of 2'-fucosyllactose in human milk. Biochem Biophys Res Commun. (1967) 28:50–3. 10.1016/0006-291X(67)90404-4 6049848

[38] Kelly RJ Rouquier S Giorgi D Lennon GG Lowe JB. Sequence and expression of a candidate for the human secretor blood group alpha(1,2)fucosyltransferase gene (FUT2). homozygosity for an enzyme-inactivating nonsense mutation commonly correlates with the non-secretor phenotype. J Biol Chem. (1995) 270:4640–9. 10.1074/jbc.270.9.4640 7876235

[39] Hegar B Wibowo Y Basrowi RW Ranuh RG Sudarmo SM Munasir Z The role of two human milk Oligosaccharides, 2'-Fucosyllactose and Lacto-N-Neotetraose, in infant nutrition. Pediatr Gastroenterol Hepatol Nutr. (2019) 22:330–40. 10.5223/pghn.2019.22.4.330 31338308 PMC6629589

[40] Kobata A. Structures and application of oligosaccharides in human milk. Proc Jpn Acad Ser B Phys Biol Sci. (2010) 86:731–47. 10.2183/pjab.86.731 20689231 PMC3066539

[41] Kunz C Meyer C Collado MC Geiger L García-Mantrana I Bertua-Ríos B Influence of gestational age, secretor, and lewis blood group status on the oligosaccharide content of human milk. J Pediatr Gastroenterol Nutr. (2017) 64:789–98. 10.1097/MPG.0000000000001402 27602704

[42] McGuire MK Meehan CL McGuire MA Williams JE Foster J Sellen DW What’s normal? Oligosaccharide concentrations and profiles in milk produced by healthy women vary geographically. Am J Clin Nutr. (2017) 105:1086–100. 10.3945/ajcn.116.139980 28356278 PMC5402033

